# Effect of nationwide reimbursement of real-time continuous glucose monitoring on HbA1c, hypoglycemia and quality of life in a pediatric type 1 diabetes population: The RESCUE-pediatrics study

**DOI:** 10.3389/fped.2022.991633

**Published:** 2022-10-06

**Authors:** Francesca De Ridder, Sara Charleer, Seppe Jacobs, Nancy Bolsens, Kristien J. Ledeganck, Sara Van Aken, Jesse Vanbesien, Inge Gies, Kristina Casteels, Guy Massa, Philippe A. Lysy, Karl Logghe, Marie-Christine Lebrethon, Sylvia Depoorter, Pieter Gillard, Christophe De Block, Marieke den Brinker

**Affiliations:** ^1^Laboratory of Experimental Medicine and Pediatrics (LEMP) and Member of the Infla-Med Center of Excellence, Faculty of Medicine and Health Science, University of Antwerp, Antwerp, Belgium; ^2^Department of Endocrinology-Diabetology-Metabolism, Antwerp University Hospital (UZA), Antwerp, Belgium; ^3^Fund for Scientific Research (FWO), Brussels, Belgium; ^4^Department of Endocrinology, University Hospitals Leuven, Catholic University of Leuven (KU Leuven), Leuven, Belgium; ^5^Department of Pediatrics, University Hospital Ghent, Ghent, Belgium; ^6^Department of Pediatrics, University Hospital Brussels, Free University of Brussels (VUB), Brussels, Belgium; ^7^Department of Pediatrics, University Hospitals Leuven, Leuven, Belgium; ^8^Department of Development and Regeneration, KU Leuven, Leuven, Belgium; ^9^Department of Pediatrics, Jessa Hospital, Hasselt, Belgium; ^10^Department of Pediatrics, University Hospital Saint-Luc, Brussels, Belgium; ^11^Department of Pediatrics, General Hospital Delta, Roeselare, Belgium; ^12^Department of Pediatrics, University Hospital of Liège, Liège, Belgium; ^13^Department of Pediatrics, General Hospital Sint-Jan Bruges, Bruges, Belgium; ^14^Department of Pediatrics, Antwerp University Hospital (UZA), Antwerp, Belgium

**Keywords:** type 1 diabetes, real-time continuous glucose monitoring (RT-CGM), HbA1c, hypoglycemia, quality of life, time in range

## Abstract

**Objective:**

Real-time continuous glucose monitoring (RT-CGM) can improve metabolic control and quality of life (QoL), but long-term real-world data in children with type 1 diabetes (T1D) are scarce. Over a period of 24 months, we assessed the impact of RT-CGM reimbursement on glycemic control and QoL in children/adolescents with T1D treated with insulin pumps.

**Research design and methods:**

We conducted a multicenter prospective observational study. Primary endpoint was the change in HbA1c. Secondary endpoints included change in time in hypoglycemia, QoL, hospitalizations for hypoglycemia and/or ketoacidosis and absenteeism (school for children, work for parents).

**Results:**

Between December 2014 and February 2019, 75 children/adolescents were followed for 12 (*n* = 62) and 24 months (*n* = 50). Baseline HbA1c was 7.2 ± 0.7% (55 ± 8mmol/mol) compared to 7.1 ± 0.8% (54 ± 9mmol/mol) at 24 months (*p* = 1.0). Participants with a baseline HbA1c ≥ 7.5% (*n* = 27, mean 8.0 ± 0.3%; 64 ± 3mmol/mol) showed an improvement at 4 months (7.6 ± 0.7%; 60 ± 8mmol/mol; *p* = 0.009) and at 8 months (7.5 ± 0.6%; 58 ± 7mmol/mol; *p* = 0.006), but not anymore thereafter (endpoint 24 months: 7.7 ± 0.9%; 61 ± 10mmol/mol; *p* = 0.2). Time in hypoglycemia did not change over time. QoL for parents and children remained stable. Need for assistance by ambulance due to hypoglycemia reduced from 8 to zero times per 100 patient-years (p = 0.02) and work absenteeism for parents decreased from 411 to 214 days per 100 patient-years (*p* = 0.03), after 24 months.

**Conclusion:**

RT-CGM in pump-treated children/adolescents with T1D showed a temporary improvement in HbA1c in participants with a baseline HbA1c ≥ 7.5%, without increasing time in hypoglycemia. QoL was not affected. Importantly, RT-CGM reduced the need for assistance by ambulance due to hypoglycemia and reduced work absenteeism for parents after 24 months.

**Clinical trial registration:**

[ClinicalTrials.gov], identifier [NCT02601729].

## Introduction

An estimated 600,900 children aged under 15 years and 1,110,100 children and adolescents (aged under 20) are living with type 1 diabetes (T1D) worldwide (1). T1D is a lifelong condition and proper management by frequent glucose monitoring and strict insulin titration is needed to avoid both hypo- and hyperglycemic events, diabetic ketoacidosis and to prevent chronic complications (2). Severe hypoglycemia affects quality of life (QoL) of children with diabetes and their parents as it may introduce fear of hypoglycemia (3), this may hinder strict glycemic control in the long term. On the other hand, hyperglycemia acutely affects brain function (4) and early diabetic ketoacidosis (DKA) exposure is associated with lower cognitive scores and altered brain growth (5). However, obtaining optimal glycemic control remains challenging despite recent advancements in insulin formulations, continuous subcutaneous insulin infusion (CSII) therapy and continuous glucose monitoring technology. Nowadays, more and more people living with diabetes have access to real-time continuous glucose monitoring (RT-CGM) which offers the ability to improve glucose control and QoL, as shown in randomized controlled and observational trials (6–9). Sensor-augmented pump (SAP) therapy, an add-on of RT-CGM on CSII therapy, allows patients to achieve stricter glucose control without increasing the risk of hypoglycemia (10, 11). This is accomplished by interrupting insulin delivery when glucose values reach or drop below a specified threshold [Low-Glucose Suspend (LGS) system] or even before hypoglycemia is reached (predictive LGS) (12). Eleven observational studies and five randomized controlled trials have been performed in children (10, 13–28), demonstrating improvements in HbA1c (10, 14–16, 19, 23, 24), in hypoglycemia (16–20, 25) and QoL (13, 15, 21, 22, 27), but data on the real-world impact of SAP in children for a period of more than 12 months are scarce (23).

Since September 2014, RT-CGM has been introduced in Belgium by means of a reimbursement program for almost 600 adults and children with T1D who use CSII and are treated in specialized diabetes centers. The effects of this reimbursement program on diabetes control in adults (*n* = 515) were evaluated in the real-life RESCUE study (Reimbursement Study of Continuous Glucose Monitoring in Belgium) and demonstrated sustained improvements in levels of HbA1c, a significant decrease in hospitalizations for hypoglycemia and ketoacidosis and work absenteeism. An important gain in quality of life presented with a strong decline in fear of hypoglycemia, particularly in those with impaired awareness of hypoglycemia (29, 30). The RESCUE study also included 75 children treated with CSII. Over a period of 24 months, this prospective observational RESCUE-Pediatrics study aimed to assess the impact of nationwide reimbursement of RT-CGM in CSII-treated children/adolescents with T1D on HbA1c levels, hypoglycemia, quality of life, hospitalization rate for severe hypoglycemia and diabetic ketoacidosis and acute events due to hypoglycemia.

## Research design and methods

### Study design

This is a 24-month real-world, prospective, observational, multi-center cohort study in 9 pediatric diabetes centers, conducted from December 2014 to February 2019, including children and adolescents (<18 years old) with T1D who received full reimbursement to start with RT-CGM. Centers were free in choosing people with T1D on CSII who they thought would benefit most from RT-CGM reimbursement, but minimum criteria for inclusion were being diagnosed with T1D > 1 year, using CSII therapy > 6 months, and willing to use RT-CGM (as a patient and as parents). Patients were expected to use RT-CGM > 70% of time and upload their RT-CGM data monthly to further receive reimbursement, instructions were given to calibrate their RT-CGM at the correct timepoints. Consecutive inclusion in the RESCUE study was done for every patient who entered the reimbursement program, after obtaining informed consent from the parents. Collaborating centers and number of patients contributed by each center can be found in Supplementary Table 1. Reimbursement was granted for an initial period of 3 years as a pilot program. The centers were legally obliged to prospectively assess the impact on clinical outcome parameters, including QoL. The study was conducted in accordance with the Declaration of Helsinki and the International Conference on Harmonization/Good Clinical Practice Guidelines and was approved by the institutional review boards and independent ethics committees of the participating centers. The study is registered at ClinicalTrials.gov (NCT02601729).

### Outcomes

The primary endpoint was the evolution of HbA1c levels over time from baseline to 24 months after reimbursement of RT-CGM. Secondary endpoints were evolutions in time spent in different glycemic ranges (Time in Range [TIR, 70–180 mg/dL, 3.9–10.0 mmol/L], time < 70 mg/dL [ < 3.9 mmol/L], time < 54 mg/dL [ < 3.0 mmol/L], time > 180 mg/dL [>10.0 mmol/L], time > 250 mg/dL [>13.9 mmol/L]) and QoL of the children and their parents. Other exploratory outcomes were demographic and social characteristics, indications for RT-CGM, and reasons for discontinuation, effect of RT-CGM on number of hospitalizations due to hypoglycemia and/or ketoacidosis, on acute events (e.g., seizures due to hypoglycemia, hypoglycemic coma, assistance by an ambulance) and on school and work (parents) absenteeism. We also explored differences between certain subgroups (based on metabolic control with a cut-off of HbA1c of 7.5%; based on pubertal status [mean age of puberty for girls was 11.2 years and for boys 11.6 years], and self-reported [by the patient] impaired hypoglycemia awareness). Impaired hypoglycemia awareness was defined as never experiencing symptoms or only at values < 54 mg/dL (<3.0 mmol/L).

### Data collection

Pre-defined clinical and demographic data were collected from a period of 12 months before until 24 months after start of the reimbursement program. Information about clinical parameters was collected from electronic clinical files at baseline, 4, 8, 12, and 24 months after start. Height was measured to the nearest millimeter using a standing stadiometer and weight was recorded to the nearest 0.1 kilogram using an electronic scale; and body mass index (BMI) was calculated. Standard deviation scores (SDS) of height, weight and BMI were calculated, using the Flemish growth study as a reference population (31). HbA1c levels were averaged for pre-specified time points: pre-reimbursement/baseline (before = −12 months until −1 day), 4 months (± 2 months), 8 months (± 2 months), 12 months (± 2 months), and 24 months (± 2 months) after start of reimbursement. RT-CGM data could only be collected after start of reimbursement, therefore data of the first 2 weeks of RT-CGM reimbursement were used to evaluate the evolution over time. RT-CGM data were collected using the designated diabetes management software from the different manufacturers (Carelink for Medtronic RT-CGM and pumps, Roche Accu-Chek Smart Pix for Roche Accu-Chek pumps or Clarity for Dexcom RT-CGM).

### Quality of life assessment

Questionnaires and standardized diaries were completed at baseline, 12 (±2), and 24 (±2) months, and scored manually (29). For school or work absenteeism, all diabetes-related reasons were taken into account, except for routine ambulatory visits with the diabetes team. Patient-reported emergency room admissions and hospitalizations for hypoglycemia and/or proven ketoacidosis were validated using hospital records in the individual centers along with other disease-related events and complications. Questionnaires used for children included the Diabetes Quality of Life for Youth (DQOLY) questionnaire (32). The DQOLY assesses the psychosocial impact of intensive diabetes regimens. It is composed of three related subscales including a 17-items diabetes life and treatment satisfaction scale, a 23-items disease impact scale, and a 13-items disease-related worries scale and a general self-rating scale of overall health. Questions were scored using a five-point Likert scale, except the question about health perception, which was scored using a four-point Likert scale. For the impact and worry scale, lower scores represent a higher quality of life, whereas for the satisfaction and health perception scale, higher scores represent a higher quality of life. For each subscale, items were summed (32). A second questionnaire was fulfilled by the parents, a part of the HAPPI-D QOL Protocol (Hvidøre, Adolescent, Parent, Professional, Instrument, Diabetes) (33). This questionnaire consisted of several components including a nine-items scale, which evaluated diabetes-related family burden regarding medical treatment, restrictions (e.g., social difficulties due to the diabetes of their child), family disruption, physical and psychological problems, and long-term health concerns. There were also questions related to altered school performance, general health of the patient and family situation (job and living situation of both parents). For the latter questions, one point was allocated if the parents were divorced, one if the mother was unemployed and one if the father was unemployed. All other questions were scored using a five-point Likert scale and summed together, with a lower score indicating a better health-related quality of life (33).

### Statistical analyses

Statistical analyses were performed using IBM SPSS Statistics version 27 and JMP Pro 15 software. Descriptive statistics were used to analyze population characteristics. Distributions of continuous data were tested for normality with the Kolmogorov–Smirnov test. Data are expressed as mean ± standard deviation or median and interquartile range (IQR). Statistical significance was defined as *p* < 0.05.

Linear mixed models were used to analyze the longitudinal data (HbA1c, CGM-glucometrics, hospitalizations, acute events, absenteeism) with a random effect of case number and a fixed effect of time. By using linear mixed models, cases with missing data still contributed to the analyses. A dichotomous variable was created to describe the number of patients that reached the ADA target HbA1c (<7.0%). The McNemar test was used to investigate the evolution over time for this variable.

## Results

### Patient characteristics

Between December 2014 and February 2019, 75 children or adolescents with T1D receiving RT-CGM reimbursement were included. Over this period, 72 (96%), 68 (91%), 62 (83%), and 50 patients (67%) used RT-CGM for 4, 8, 12, and 24 months, respectively.

Baseline characteristics are shown in Table 1. Most participants were male (57%). The mean age was 9.5 ± 4.3 years, diabetes duration 5.0 ± 3.6 years and pump duration 3.5 ± 2.7 years. Thirty children (40%) were already in their puberty at the time of inclusion. The mean HbA1c at baseline was 7.2 ± 0.7%. Thirty-two children (43%) presented with impaired hypoglycemia awareness (IHA). The majority of children had limited experience with prior RT-CGM use, but 13 children were already using RT-CGM for >4 months prior to reimbursement. The most important reasons to start with RT-CGM were a young age, IHA, living conditions such as a variable lifestyle (of the child) or a very unstable glucose control (see Table 1). Parents of the children with T1D were mostly highly educated.

**TABLE 1:** Baseline characteristics of pediatric patients in the real-time continuous glucose monitoring (RT-CGM) reimbursement program.

General characteristics	
Children (n)	75
Gender (female)	32 (43)
Ethnicity (Caucasian)	75 (100)
Age (years)	9.5 ± 4.3
Height (cm)	135.4 ± 26.5
Height (SDS)	−0.17 ± 1.06
Weight (kg)	34.0 ± 15.1
Weight (SDS)	0.22 ± 0.99
BMI (kg/m^2^)	17.5 ± 2.1
BMI (SDS)	0.18 ± 0.98
**Education**	
Parents of children*	
Higher (everything beyond secondary education)	62 (83)
No higher	13 (17)
**Diabetes-related characteristics**	
Diabetes duration (years)	5.0 ± 3.6
Age at diagnosis (years)	4.2 ± 3.0
Duration of insulin pump therapy (years)	3.5 ± 2.7
Impaired hypoglycemia awareness (yes)	28 (38)
**RT-CGM–related Characteristics**	
**Reasons for RT-CGM therapy†**	
Poor control without good explanation	10 (13)
Impaired hypoglycemia awareness	32 (43)
Frequent severe hypoglycemic events	9 (12)
Epilepsy with hypoglycemia	7 (9)
Ketoacidosis	2 (3)
Very unstable blood glucose control (not further specified)	17 (23)
To improve adherence	5 (7)
Sport	14 (19)
Living conditions (varying life of the child)	28 (37)
Age	50 (67)
Other	25 (33)
**Prior RT-CGM usage**	
No	42 (56)
Blinded	2 (3)
Sporadically	5 (7)
Continuously for ≤ 4 months	1 (1)
Continuously for >4 months	13 (17)
Test sensor	12 (16)
Prior RT-CGM usage duration (months)‡	22 [19–24]

Results are presented as n (% of patients), mean ± SD or median [IQR]. *Defined as highest education of one (=n) of two parents. †Can be more than one reason per patient. ‡For those already using RT-CGM continuously.

### Real-time continuous glucose monitoring usage and dropouts

Sixty-five (87%) participants used a Medtronic pump (Paradigm Veo 754: *n* = 38; MiniMed^®^ 640G: *n* = 23, Paradigm Veo 554: *n* = 3; and Paradigm 722: *n* = 1) and 10 children used a Roche Accu-Chek^®^ Combo insulin pump. Sensors used by patients were Medtronic MiniMed^®^ Enlite^®^ Sensor (n = 50; 67%) and Dexcom G4^®^ PLATINUM (n = 25; 33%).

The usage of the RT-CGM system was 88 ± 8% after 12 months and still 87 ± 10% after 24 months. Thirteen children discontinued RT-CGM after 12 months and another twelve children after 24 months, mostly because of an excessive amount of alarms (*n* = 7; 28% of children who stopped). Other reasons were e.g., use of the system < 70% of the time (*n* = 5; 20% of children who stopped), technical problems (n = 5; 20% of children who stopped), lack of perceived benefit (*n* = 5; 20% of children who stopped), lack of achieving personal glycemic target (*n* = 3; 12% of children who stopped), skin irritations (*n* = 2; 8% of children who stopped) or another reason (*n* = 6; 24% of children who stopped).

### Evolution of glucometrics after introduction of real-time continuous glucose monitoring reimbursement

#### Hemoglobin A1c

**FIGURE 1:**
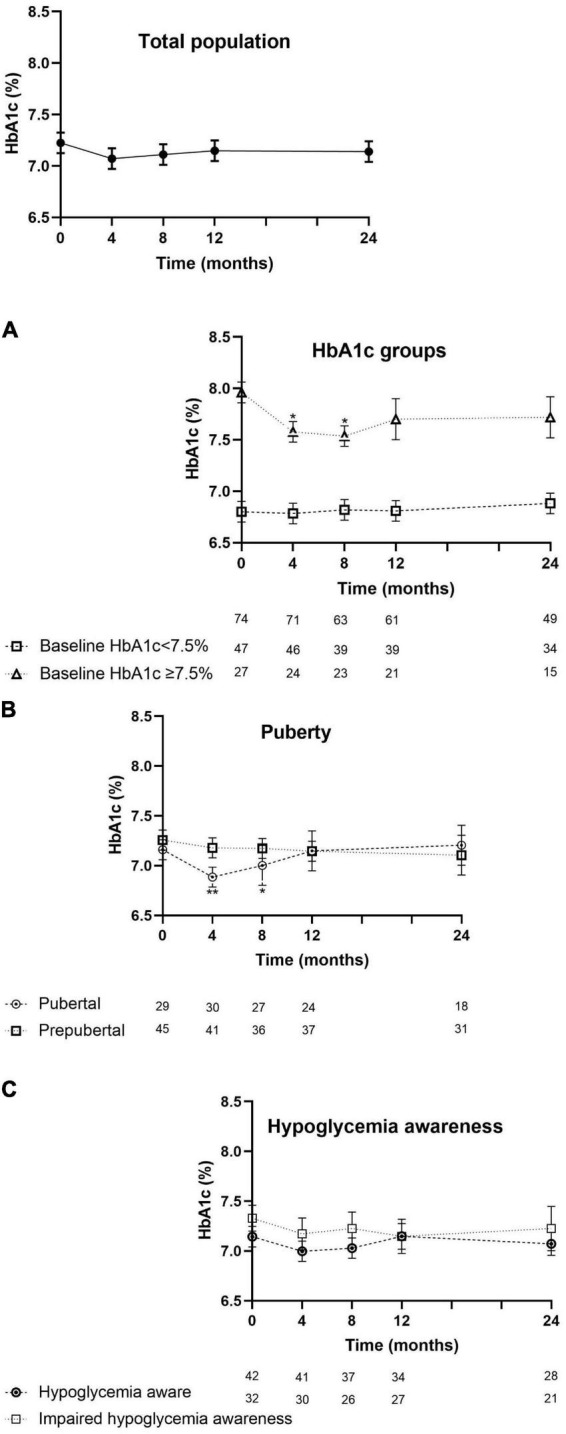
Evolution of HbA1c (mean ± SEM) from the start of the reimbursement program until 24 months later. HbA1c of the intention-to-treat population. Assessments per subpopulation: **(A)** by HbA1c level at entry. **(B)** By puberty or not pubertal yet at the start of RT-CGM therapy and **(C)** by being hypoglycemia aware or having impaired hypoglycemia awareness. Values at 4, 8, 12, and 24 months were compared with the values at baseline. Asterisks denote statistical significance for comparison, with *p < 0.05 and **p < 0.005.

The evolution of HbA1c over the 24-month period is depicted in Figure 1. For the total population, the mean HbA1c at baseline was 7.2 ± 0.7% (55 ± 8 mmol/mol) and evolved toward 7.1 ± 0.8% after 12 and 24 months (54 ± 9 mmol/mol; p = 0.5; p = 1.0) (see Supplementary Table 2).

In children with a higher baseline HbA1c (≥7.5%; *n* = 27), their mean HbA1c at start (8.0 ± 0.3%, 64 ± 3 mmol/mol) improved toward 7.6 ± 0.7% at 4 months (60 ± 8 mmol/mol; *p* = 0.009), 7.5 ± 0.6% (58 ± 7mmol/mol; p = 0.006) at 8 months, and then stabilized at 7.7 ± 0.7% at 12 months (61 ± 8mmol/mol; *p* = 0.1) and 7.7 ± 0.9% (61 ± 10 mmol/mol; *p* = 0.2) at 24 months. In the children with a baseline HbA1c < 7.5% (*n* = 47), HbA1c remained stable throughout 24 months (Figure 1A).

Sixty-three percent of the children already had an Hb1Ac < 7,5% at the start of the trial. The proportion of children who reached the new target HbA1c of <7% increased from 37% at start to 42% after 12 and 24 months (*p* = 0.8; *p* = 0.6).

In pubertal children (*n* = 30), HbA1c decreased from 7.1 ± 0.7% (54 ± 8 mmol/mol) at baseline to 6.8% ± 0.7% at 4 months (51 ± 8 mmol/mol; *p* = 0.001) and to 7.0 ± 0.8% at 8 months (53 ± 9 mmol/mol; *p* = 0.04) and returned back to 7.1 ± 0.8% (54 ± 9 mmol/mol) at 12 months and thereafter. In the prepubertal subgroup (*n* = 45), the mean baseline HbA1c did not significantly change (Figure 1B). The evolution of HbA1c over time was not influenced by hypoglycemia awareness status (Figure 1C).

#### Time in different glucose ranges

**FIGURE 2:**
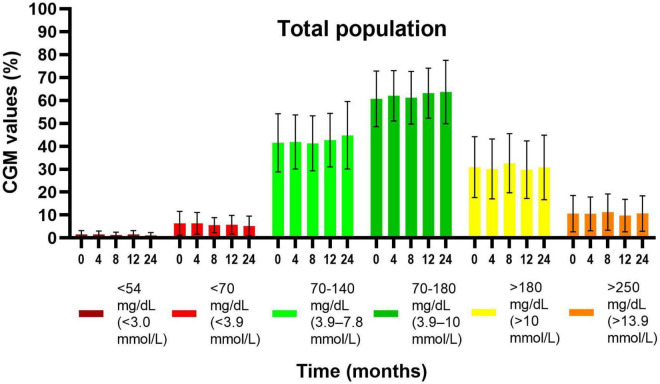
Evolution of glycemic values by CGM readings (mean ± SD) from the first 2 weeks of the reimbursement program until 24 months later, in the total population. Values at 4, 8, 12, and 24 months were compared with the values at baseline (first 2 weeks). There were no significant differences in time.

For the total population, TIR was 60.7 ± 12.2% in the first 2 weeks, 63.2 ± 10.9% and 63.7 ± 13.8% after 12 and 24 months, respectively, there were no significant changes (Figure 2). For children with baseline HbA1c of < 7.5%, TIR was significantly higher at 24 months (68.0 ± 12.4%; *p* = 0.009) compared to the first 2 weeks (63.5 ± 13.5%). Their time spent between 70 and 140 mg/dL improved from 45.1 ± 13.9% to 49.4 ± 14.2% (*p* = 0.03). On the other hand, no significant differences in TIR were observed for children with baseline HbA1c ≥ 7.5%. TIR was higher in pubertal children (63.0 ± 14.5%) compared to prepubertal (58.9 ± 9.9%) in the first 2 weeks and showed an overall trend toward improvement in both groups. Time between 70 and 140 mg/dl can be found in Supplementary Table 3. TIR values were comparable between children with or without IHA.

Time < 70 mg/dL (<3.9 mmol/L) and < 54 mg/dL (<3.0 mmol/L) did not change significantly over 24 months (Figure 2). There were no significant differences in evolution of time spent in hypoglycemia between subgroups (see Supplementary Table 3).

Time > 180 mg/dL (>10.0 mmol/L) and >250 mg/dL (>13.9 mmol/L) did not change over time (Figure 2), both in the total population and between subgroups (see Supplementary Table 3).

#### Consensus targets for the different glucose ranges

The clinical targets for continuous glucose monitoring as proposed by Battelino et al. were investigated (34). The recommended TIR of >70% was achieved by 13% of the total group of children at start and in 26% after 12 and 24 months (*p* = 0.1; *p* = 0.2).

Consensus targets for hypoglycemia < 70 mg/dL (<3.9 mmol/L) of <4% of the time, were reached by 27% of the total group at start, in 35% after 12 months (*p* = 0.6) and 42% after 24 months (*p* = 0.03). Targets for hypoglycemia < 54 mg/dL (<3.0 mmol/L) of <1% of the time, were reached by 35% of the total group at start, in 40% after 12 months (*p* = 0.4) and 46% after 24 months (*p* = 0.5).

Consensus targets for hyperglycemia > 180 mg/dL (>10.0 mmol/L) of <25% of the time, were achieved by 25% of the total group at start, in 34% after 12 months (*p* = 0.4) and 34% after 24 months (*p* = 0.2). Targets for hyperglycemia > 250 mg/dL (>13.9 mmol/L) of <5% of the time, were achieved by 16% of the total group at start, in 29% after 12 months (*p* = 0.1) and 20% after 24 months (*p* = 1.0).

### Glycemic variability

Glycemic variability as assessed by standard deviation (SD) and coefficient of variation (CV) of RT-CGM data also remained stable over 24 months (SD: 66 ± 15 mg/dl in the first 2 weeks vs. 67 ± 13 mg/dl at 24 months; CV: 42 ± 6% in the first 2 weeks vs. 43 ± 5% at 24 months). Only 10 children (13%) had a CV of ≤36% in the first 2 weeks and only 4 (8%) at 24 months.

### Hospitalizations and days of absenteeism

In the year prior to reimbursement of RT-CGM, there were 8 hospitalizations per 100 patient-years (py) due to hypoglycemia and 4 hospitalizations per 100 py because of DKA. After 12 months, there were 2 hypoglycemic (*p* = 0.2) and 2 DKA hospitalizations per 100 py (*p* = 0.9). After 24 months, 4 hospitalizations per 100 py occurred due to hypoglycemia (*p* = 0.5) and 6 per 100 py due to ketoacidosis (*p* = 0.9).

Severe hypoglycemic events (with seizures, coma, ambulance) only occurred in the children with IHA. At start, there were 22 hypoglycemic events with seizure per 100 py in the total group, 6 events per 100 py occurred after 12 months (*p* = 0.5) and there were no seizures anymore after 24 months (*p* = 0.2). At start, there were 16 hypoglycemic events with seizure in total with one child having 10 attacks. Hypoglycemic coma occurred 4 times per 100 py prior to reimbursement and it did not occur anymore at the 12- and 24-month time points (*p* = 0.3). Assistance from an ambulance due to hypoglycemia was needed 8 times per 100 py. After 12 months, this occurred in 2 occasions per 100 py (*p* = 0.07). After 24 months, assistance from an ambulance was no longer required (*p* = 0.02).

School absenteeism consisted of 274 days at baseline, 267 days after 12 months (*p* = 0.4) and 218 days per 100 py after 24 months (*p* = 0.09). Parents were absent from work for 411 days at baseline, 398 days at 12 months (*p* = 0.3) with a significant decrease to 214 days per 100 py after 24 months (*p* = 0.03).

### Evolution of quality of life after introduction of real-time continuous glucose monitoring

In the children/adolescents, DQOLY scores did not change over time. The mean scores at start were 53 ± 10 for the category “impact,” 69 ± 9 for “satisfaction” and 19 ± 8 for “worry.” After 12 and 24 months, we did not find any significant changes in these different categories, neither between subgroups.

The total score of QoL of parents was 21 ± 6 at the start of RT-CGM-reimbursement and did not change after 12 and 24 months.

## Discussion

Although the clinical benefits of RT-CGM have been demonstrated in randomized controlled trials (RCTs) before, they often only analyzed 6- or 12-month data or even less (10, 13–19, 21, 22, 24), and therefore do not inform about sustainability of its effects. Furthermore, most RCTs do not assess the effect of novel technology on hospitalizations because being hospitalized due to hypoglycemia or ketoacidosis is usually an exclusion criterion. Therefore, real-world data are needed to evaluate long-term effectiveness of technology like the add-on of RT-CGM and SAP.

The RESCUE-Pediatrics study is a 24-month, real-life observational study, evaluating the implementation of a national reimbursement system for RT-CGM in 75 children and adolescents using an insulin pump. Children/adolescents in this RESCUE-Pediatrics study had a good baseline HbA1c, were highly motivated and sensor usage was very high during the entire study, which all contributed to the results. The motivation was probably driven by the knowledge that reimbursement was solely continued in patients who used RT-CGM > 70% of time.

Data on SAP in a pediatric population studying the effect on HbA1c, hypoglycemia and/or QoL, are emerging (10, 13–24, 26–28). Eleven observational studies and five RCTs introducing RT-CGM in pediatric pump users, have been performed from 2010 until mid-2022, including 16 to 622 (of which 129 on SAP) children/adolescents with T1D per study, with a trial duration of 2 to 36 months. The following studies compared PLGS or LGS vs. SAP without extra features, or SAP vs. CSII with intermittently scanned CGM (isCGM) or SMBG, or SAP vs MDI. Two RCTs compared (hybrid) closed-loop control to SAP (25, 35). Main limitations of these studies are a rather short follow-up [only two studies with a follow-up longer than 12 months (23, 28)] and small study groups (nine studies with less than 50 patients).

Seven studies showed improvement in HbA1c with SAP from 0.3 to 0.7%, with baseline HbA1c ranging between 7.6 and 8.4% (10, 14–16, 19, 23, 24). Seven studies showed stable HbA1c values (17, 18, 25–28, 35) or only improvement in patients with a poorer metabolic control at start (HbA1c > 7% of >7.5%) (21, 22). The real-world study of Scaramuzza et al. including 622 children with T1D with a follow-up of up to 36 months, compared SAP versus CSII (23). A greater reduction in HbA1c with SAP (0.6%, 7mmol/mol) compared to CSII (0.3%,

3mmol/mol) and a reduction in the number of severe hypoglycemic events with SAP was observed.

At baseline, the RESCUE-Pediatrics cohort had a good mean HbA1c close to the recommended HbA1c levels for good metabolic control, according to the ISPAD guidelines (36). RT-CGM resulted in a stable HbA1c over 24 months in the total cohort, but in those with a higher baseline HbA1c, a temporary significant improvement of maximum 0.5% at 8 months was achieved, without increasing time in hypoglycemia. We hypothesize that the initial reduction in HbA1c after 4–8 months is likely due to an augmented focus on correct treatment and lifestyle at the start of the study. Continuous stimulation and reinforcing are needed from the medical team in order to maintain the beneficial RT-CGM effect in a pediatric/adolescent population.

In those with a good baseline HbA1c, HbA1c remained stable while TIR improved from 63.5 to 68.0% at 24 months. The improvement in TIR without an amelioration in HbA1c may be explained by the definition and thresholds of TIR. A small decrease in both time below and time above range might increase TIR, without affecting mean glycemia or HbA1c. TIR was higher in pubertal children compared to prepubertal children, which is an exceptional result and shows that this cohort had highly motivated pubertal children, not a representative group of adolescents.

Thirty-seven percent of the children were already well-controlled according to a baseline HbA1c of <7%, 63% had an Hb1Ac < 7.5%, and seventeen percent had already used RT-CGM continuously before entering this study. These are possible reasons why there was not always a great improvement by RT-CGM therapy on the different parameters.

Time in hypoglycemia in the RESCUE-Pediatrics study was low in the first 2 weeks without significant changes throughout the 24-month follow-up period. Results on hypoglycemia reported vary between studies. Hypoglycemia < 70 mg/dL and hypoglycemia < 54 mg/dL improved greatly in one study with a reduction in percentage of 5.9% (from 7.4 to 1.5%) and 2.6% (from 2.8 to 0.2%), respectively (16). One study showed a 3% lower rate of hypoglycemia < 65 mg/dL (3.6 mmol/L) with PLGS, compared to CSII with RT-CGM without automated insulin suspension (20). Four studies showed a significant improvement in hypoglycemia < 70 mg/dL of less than 2% and/or an improvement of less than 1% in hypoglycemia < 54 mg/dL (17–19, 25). One study showed an increase in hypoglycemia of 0.8% < 70 mg/dL (from 1.0 to 1.8%) and 0.2% < 54 mg/dL (from 0.1 to 0.3%) (35).

Less diabetes-related hospital admissions (25.9 vs. 13.9 per 100 patients-years [*p* = 0.0002]) and a shorter length of stay (1.7 ± 5.0 vs. 0.6 ± 2.6 days [*p* < 0.001]) after 12 months of SAP-therapy was reported in the INTERPRET study (21). Two other studies reported no severe hypoglycemic or diabetic ketoacidosis events (19, 35). In the study of Scaramuzza et al., no diabetic ketoacidosis episodes were observed during the follow-up, and severe hypoglycemia significantly decreased in SAP patients (23).

In our RESCUE-Pediatrics study, because of the low number of participants and events, we did not observe a significant change in number of hospitalizations for ketoacidosis and hypoglycemia. The severe hypoglycemic events only occurred in children with IHA; and there were no severe events anymore after 24 months. This was not significant, probably since most of the severe events occurred in only one child. The need of assistance of an ambulance due to hypoglycemia was only needed in children with IHA and decreased significantly. These findings underscore the need for RT-CGM with alarms or automated insulin cessation in these children. We are the first to report that only children with IHA had severe hypoglycemic events. To the best of our knowledge, no comparable data from other pediatric studies are available. One of the most important factors to opt for RT-CGM is severe or recurrent hypoglycemia, or IHA. RT-CGM and SAP focus primarily on hypoglycemia avoidance, which helped in pursuing the consensus targets (34) for hypoglycemia.

Quality of life was investigated in seven studies and these reported a high treatment satisfaction, less worries about diabetes for children and their parents, less fear of hypoglycemia and/or improved sleep for parents (13, 15, 18, 21, 22, 27) or no change in quality of life (17). Our RESCUE-Pediatrics study showed no change in quality of life most likely due to the fact that these children and adolescents already had a rather high quality of life before this study, compared to the mean QoL scores from the validation study of Skinner et al. (37). Thirty-two percent of the children discontinued RT-CGM due to alarm fatigue, reflecting an increased disease burden.

The RESCUE trial originated from a unique requirement of quality control imposed by the Belgian health care authorities, linking reimbursement of RT-CGM/SAP to a pre-planned analysis of data. The teams of the different centers were free to include those patients of whom they thought would benefit most. This differs from RCTs or other real-world studies in which persistently high HbA1c is a main inclusion criterion. In addition, since the children/adolescents had to use RT-CGM for >70% of the time, this may have introduced a selection bias because the participants were motivated to use their newly introduced technology intensively. This study is not an RCT, making it possible that other factors such as training, education, and more intense contact contributed to the overall results.

Reimbursement of RT-CGM for children reduced the number of hospitalizations due to hypoglycemia, the need of ambulance assistance and transport for severe hypoglycemia, the number of days of work absenteeism and tended to reduce the number of days of school absenteeism. Taking into account the average price for one hospitalization for DKA/hypoglycemia of €4,733 (29) and an ambulance cost of €160, it represents a nationwide cost reduction of about €30,000 per year. Parental work absenteeism was reduced by 36%, corresponding to a total reduction of about €16,000 per year (calculated taking an average income of €1,842/month) (see Supplementary Table 4). These calculations did not take into account the developmental and financial beneficial effect of reduced school absenteeism. These cost reductions must be balanced against the extra costs generated by RT-CGM devices, more expensive pumps and 4 h of extra education. We did not do a formal cost efficacy study but these assumptions are valid for our population with already a rather good metabolic control, but are probably even more convincing in participants with worse metabolic control.

## Conclusion

Real-time continuous glucose monitoring/SAP in children/adolescents with T1D resulted in a temporary improvement in those with a high baseline HbA1c of ≥7.5%, without increasing time in hypoglycemia. We observed an important benefit in terms of need of assistance from an ambulance and days of work absenteeism for parents, which supports the rationale that RT-CGM instigates altered behavior. These effects were seen over a 24-month follow-up, making this one of the longest real-world follow-up studies.

## Author’s note

Part of this study was presented at the 12th International Conference on Advanced Technologies and Treatments for Diabetes (oral presentation), Berlin, Germany, 2019; at the Belgian Endocrine Society (oral presentation), Brussels, Belgium, 2019; and at the American Diabetes Association (poster presentation), San Francisco, United States, 2019.

## Data availability statement

The datasets presented in this article are not readily available due to patient data privacy. Requests to access the de-identified datasets should be directed to the corresponding author.

## Ethics statement

The studies involving human participants were reviewed and approved by the Ethics Committee of Antwerp University Hospital (UZA), Edegem, Belgium. Written informed consent to participate in this study was provided by the participants’ legal guardian/next of kin.

## Author contributions

FDR, SC, and SJ collected and analyzed the data, performed statistical analyses, discussed, and wrote the manuscript, and made figures and tables. MB, NB, SVA, JV, IG, KC, PG, GM, PL, KL, M-CL, and SD included the patients, collected the data, and critically reviewed the manuscript. MB, CDB, and PG designed the study, analyzed and discussed the data, and wrote the manuscript. KJL also discussed the data and edited the manuscript. FDR, SC, PG, MB, and CDB are the guarantors of this work and as such had full access to all the data in the study and takes responsibility for the integrity of the data, and accuracy of the data analysis. All authors contributed to the article and approved the submitted version.

## Abbreviations

CSII: continuous subcutaneous insulin infusion
CV: coefficient of variation
DKA: diabetic ketoacidosis
HbA1c: hemoglobin A1c
IHA: impaired hypoglycemia awareness
isCGM: intermittently scanned continuous glucose monitoring
(P)LGS: (Predictive) low-glucose suspend
MDI: multiple daily injections
py: patient-years
QoL: quality of life
RCT: randomized controlled trial
RESCUE: reimbursement study of continuous glucose monitoring in Belgium
RT-CGM: real-time continuous glucose monitoring
SAP: sensor-augmented pump
SD(S): standard deviation (Score)
SEM: standard error of the mean
SMBG: self-monitoring of blood glucose
T1D: type 1 diabetes
TIR: time in range
UZA: University Hospital of Antwerp
