# Supplementary material for: Effect of nationwide reimbursement of real-time continuous glucose monitoring on HbA1c, hypoglycemia and quality of life in a pediatric type 1 diabetes population: The RESCUE-pediatrics study
Source: Front Pediatr. 2022 Oct 6;10:991633. doi: 10.3389/fped.2022.991633 (PMC9582657; doi:10.3389/fped.2022.991633)
Supplement: Supplementary file 1 [file Table_1.DOCX]

**Supplementary Table 1**: List of participating centers

| **Departments of Endocrinology and/or Pediatrics** | **Number of children** |
| --- | --- |
| 1. Antwerp University Hospital | 29 |
| 2. University Hospital Ghent | 12 |
| 3. University Hospital Brussels, Free University of Brussels | 10 |
| 4. University Hospitals Leuven, Catholic University of Leuven | 5 |
| 5. Jessa Hospital, Hasselt | 5 |
| 6. University Hospital Saint-Luc, Brussels | 5 |
| 7. General Hospital Delta, Roeselare | 4 |
| 8. University Hospital of Liège | 3 |
| 9. General Hospital Sint-Jan Bruges | 2 |

**Supplementary table 2:** HbA1c levels at start and at different time points during 24 months of RT-CGM

|  | **At start** | **4 months** | **8 months** | **12 months** | **24 months** |
| --- | --- | --- | --- | --- | --- |
| **HbA1c in the total group** | | | | | |
| Children (n)  (%)  (mmol/mol)  p-value | 74  7.2±0.7  55±8 | 71  7.1±0.8  54±9  0.06 | 63  7.1±0.7  54±8  0.2 | 61  7.1±0.8  54±9  0.5 | 49  7.1±0.8  54±9  1.0 |
| **HbA1c at start <7.5%** | | | | | |
| Children (n)  (%)  p-value | 47  6.8±0.5 | 46  6.8±0.7  1.0 | 39  6.8±0.6  1.0 | 39  6.8±0.6  1.0 | 34  6.9±0.6  0.4 |
| **HbA1c at start ≥7.5%** | | | | | |
| Children (n)  (%)  p-value | 27  8.0±0.3 | 24  7.6±0.7  0.009* | 23  7.5±0.6  0.006* | 21  7.7±0.7  0.1 | 15  7.7±0.9  0.2 |
| **Pubertal children** | | | | | |
| Children (n)  (%)  p-value | 29  7.1±0.7 | 30  6.8±0.7  0.001** | 27  7.0±0.8  0.04* | 24  7.1±0.8  0.5 | 18  7.1±0.7  1.0 |
| **Prepubertal children** | | | | | |
| Children (n)  (%)  p-value | 45  7.3±0.7 | 41  7.2±0.7  1.0 | 36  7.2±0.7  0.9 | 37  7.2±0.8  0.9 | 31  7.2±0.9  1.0 |
| **Hypoglycemia aware children** | | | | | |
| Children (n)  (%)  p-value | 42  7.1±0.7 | 41  7.0±0.7  0.2 | 37  7.0±0.6  0.3 | 34  7.1±0.8  1.0 | 28  7.1±0.6  1.0 |
| **Children with impaired hypoglycemia awareness** | | | | | |
| Children (n)  (%)  p-value | 32  7.3±0.7 | 30  7.2±0.9  0.4 | 26  7.2±0.8  0.8 | 27  7.1±0.9  0.5 | 21  7.2±1.0  1.0 |

Results are presented as mean±SD. N is mentioned for each timepoint (missing data is truly missing or because of the drop out of a patient). Differences compared to baseline are statistically significant if *p <0.05, **p<0.005.

**Supplementary table 3:** CGM values at start (first 2 weeks) and at different time points during 24 months of RT-CGM

|  | **2 weeks** | **4 months** | **8 months** | **12 months** | **24 months** |
| --- | --- | --- | --- | --- | --- |
| **CGM values in the total group (% of time)** | | | | | |
| Children (n)  >250 mg/dL  (>13.9 mmol/L)  >180 mg/dL  (>10 mmol/L)  70–180 mg/dL  (3.9–10 mmol/L)  70–140 mg/dL  (3.9–7.8 mmol/L)  <70 mg/dL  (<3.9 mmol/L)  <54 mg/dL  (<3.0 mmol/L) | 60  10.6±7.9  30.9±13.3  60.7±12.2  41.5±12.7  6.4±5.2  1.5±1.7 | 64  10.5±7.4  30.1±13.1  62.0±11.0  41.9±11.8  6.4±4.7  1.5±1.5 | 55  11.3±7.9  32.6±12.9  61.2±11.5  41.3±12.0  5.6±3.3  1.3±1.2 | 57  9.8±7.1  29.8±12.6  63.2±10.9  42.7±11.7  5.8±4.1  1.5±1.7 | 45  10.7±7.7  30.8±14.1  63.7±13.8  44.8±14.7  5.2±4.3  1.1±1.3 |
| **HbA1c at start <7.5%** | | | | | |
| Children (n)  >250 mg/dL  >180 mg/dL  70–180 mg/dL  70–140 mg/dL  <70 mg/dL  <54mg/dL | 37  9.6±8.8  27.0±14.0  63.5±13.5  45.1±13.9  7.2±5.8  1.6±1.9 | 41  7.8±5.9  25.2±11.6  65.4±10.0  45.6±11.8  7.1±5.1  1.7±1.8 | 35  8.7±6.8  27.9±12.2  65.0±11.1  45.6±12.1  6.3±3.2  1.2±0.9 | 40  7.8±5.8  26.1±11.5  65.9±10.2  46.1±10.8  6.7±4.5  1.8±1.9 | 31  8.3±6.3  26.0±12.3  68.0±12.4*  49.4±14.2*  5.6±4.5  1.3±1.2 |
| **HbA1c at start ≥7.5%** | | | | | |
| Children (n)  >250 mg/dL  >180 mg/dL  70–180 mg/dL  70–140 mg/dL  <70 mg/dL  <54mg/dL | 21  12.3±5.9  37.6±9.3  56.5±7.8  35.8±7.5  5.1±3.5  1.2±1.4 | 22  15.0±7.7  38.8±11.1  56.0±10.3  35.5±9.1  4.6±2.6  1.0±0.9 | 20  15.8±7.7  40.9±9.7  54.4±9.0  34.0±7.6  4.3±3.2  1.4±1.6 | 17  14.4±7.8  38.7±10.9  56.9±10.0  34.7±9.8  3.9±2.2  0.8±0.7 | 14  15.9±8.1  41.6±11.9  54.1±12.0  34.5±10.3  4.1±3.7  0.7±1.4 |
| **Pubertal children** | | | | | |
| Children (n)  >250 mg/dL  >180 mg/dL  70–180 mg/dL  70–140 mg/dL  <70 mg/dL  <54mg/dL | 27  9.2±8.7  28.3±14.9  63.0±14.5  44.5±14.0  6.9±5.8  1.6±1.9 | 27  9.0±7.7  26.3±13.2  64.9±11.6  45.4±11.4  7.1±5.7  1.7±1.9 | 20  11.9±9.2  32.8±14.6  61.7±13.7  40.9±13.7  4.9±2.5  1.0±0.7 | 22  9.0±8.5  28.0±14.7  65.2±12.9  44.6±13.0  5.7±5.0  1.7±2.3 | 18  9.6±9.4  28.1±16.8  67.3±17.8  50.0±18.9*  4.3±4.4  1.0±1.4 |
| **Prepubertal children** | | | | | |
| Children (n)  >250 mg/dL  >180 mg/dL  70–180 mg/dL  70–140 mg/dL  <70 mg/dL  <54mg/dL | 33  11.7±7.0  32.9±11.8  58.9±9.9  39.2±11.2  5.9±4.6  1.4±1.5 | 37  11.6±7.2  32.9±12.5  59.9±10.1  39.2±11.6  5.8±3.7  1.3±1.2 | 35  11.0±7.1  32.6±12.0  60.8±10.3  41.5±11.1  6.0±3.7  1.4±1.4 | 35  10.4±6.0  31.0±11.2  61.9±9.3  41.4±10.7  5.9±3.5  1.4±1.3 | 27  11.4±6.4  32.7±12.0  61.3±10.1  41.4±10.2  5.7±4.2  1.2±1.3 |
| **Hypoglycemia aware children** | | | | | |
| Children (n)  >250 mg/dL  >180 mg/dL  70–180 mg/dL  70–140 mg/dL  <70 mg/dL  <54mg/dL | 34  10.7±8.7  30.2±13.8  61.7±13.0  42.6±12.7  5.7±4.5  1.3±1.3 | 36  9.4±6.7  28.1±11.9  64.0±10.3  43.3±10.9  5.7±4.0  1.3±1.4 | 29  11.2±7.6  32.4±11.8  61.6±10.9  40.9±10.7  5.3±3.5  1.3±1.4 | 32  8.8±6.3  28.5±12.3  65.1±11.1  44.2±11.7  4.7±2.7  1.4±1.6 | 24  10.3±7.5  30.3±14.7  65.2±14.9  46.2±16.7  4.2±4.0  0.9±1.2 |
| **Children with impaired hypoglycemia awareness** | | | | | |
| Children (n)  >250 mg/dL  >180 mg/dL  70–180 mg/dL  70–140 mg/dL  <70 mg/dL  <54mg/dL | 25  10.4±6.8  31.8±12.9  59.4±11.1  40.1±12.8  7.2±5.9  1.7±2.1 | 28  11.8±8.2  32.7±14.4  59.5±11.4  40.1±12.9  7.2±5.4  1.6±1.7 | 26  11.5±8.3  32.9±14.2  60.7±12.4  41.7±13.5  5.9±3.2  1.3±0.9 | 25  11.1±7.8  31.5±13.0  60.7±10.2  40.8±11.6  7.2±5.1  1.7±1.9 | 21  11.1±8.1  31.4±13.8  62.0±12.6  43.2±12.4  6.3±4.4  1.3±1.4 |

Results are presented as mean±SD. N is mentioned for each timepoint (missing data is truly missing or because of the drop out of a patient). Differences compared to baseline (first two weeks) are statistically significant if *p<0.05.

**Supplementary table 4:** Evolution of Diabetes-Related Hospital Admissions and Work Absenteeism

|  | **Before reimbursement**  **(n = 75)** | **12m of reimbursement**  **(n=58)** | **24m of reimbursement**  **(n=48)** | **P-value**  **12/24m** |
| --- | --- | --- | --- | --- |
| **Absolute numbers** |  |  |  |  |
| Hospitalizations due to hypoglycemia | 6 | 1 | 2 | 0.2  0.5 |
| Hospitalizations due to ketoacidosis | 3 | 1 | 3 | 0.9  0.9 |
| Ambulance assistance for severe hypoglycemia | 6 | 1 | 0 | 0.07  0.02* |
| School absenteeism | 205 | 165 | 109 | 0.4  0.09 |
| Work absenteeism^**^ | 308 | 247 | 107 | 0.3  0.03* |
| **Number per 100 patient years** |  |  |  |  |
| Hospitalizations due to hypoglycemia | 8 | 2 | 4 | 0.2  0.5 |
| Hospitalizations due to ketoacidosis | 4 | 2 | 6 | 0.9  0.9 |
| Ambulance assistance for severe hypoglycemia | 8 | 2 | 0 | 0.07  0.02* |
| School absenteeism | 274 | 267 | 218 | 0.4  0.09 |
| Work absenteeism^**^ | 411 | 398 | 214 | 0.3  0.03* |

Patient-reported hospital admissions were validated by clinicians. Differences after 12 and 24 months (12/24m) compared to baseline are statistically significant if *p<0.05.

**Work absenteeism of at least half a day.
